# Impact of different clear corneal incision sizes on anterior corneal aberration for cataract surgery

**DOI:** 10.5935/0004-2749.20200089

**Published:** 2024-02-11

**Authors:** Qing-Qing Tan, Jing Tian, Xuan Liao, Jia Lin, Bai-Wei Wen, Chang-Jun Lan

**Affiliations:** 1 Department of Ophthalmology, Affiliated Hospital of North Sichuan Medical College; Department of Ophthalmology and Optometry, North Sichuan Medical College, Nanchong, China

**Keywords:** Phacoemulsification, Astigmatism, Cornea/surgery, Surgical wound, Treatment outcome, Facoemuslificação, Astigmatismo, Cornea/cirurgia, Ferida cirúrgica, Resultado de tratamento

## Abstract

**Purpose:**

To investigate the impact of different sizes of steep meridian clear corneal incisions for phacoemulsification on anterior corneal higher-order aberrations.

**Methods:**

Medical records of patients who underwent 2.2-mm coaxial micro-incision cataract surgery or 2.75-mm coaxial small-incision cataract surgery were retrospectively reviewed. Only patients with preexisting anterior corneal astigmatism <2.00 diopters (D) and ≥0.50 D who underwent a steep meridian clear corneal incision were included. Primary outcomes were 3^rd^- to 6^th^-order anterior corneal higher-order aberrations with an 8-mm pupil. Anterior corneal astigmatism and effective phaco time were evaluated as secondary outcomes. Preoperative and 3-month postoperative outcomes were evaluated.

**Results:**

Anterior corneal astigmatism significantly decreased after both procedures; however, there was no significant difference found in surgically induced anterior corneal astigmatism between the two procedures (p=0.146). Although the total higher-order aberrations did not significantly change after both procedures, the group comparison showed a significant difference in surgically induced total higher-order aberrations (a decrease of 0.337 ± 1.156 µm in 2.2-mm coaxial micro-incision cataract surgery and an increase of 0.106 ± 0.521 µm in 2.75-mm coaxial small-incision cataract surgery, p=0.046). Spherical aberrations significantly decreased after 2.2-mm coaxial micro-incision cataract surgery (p=0.001), whereas they did not change significantly after 2.75-mm coaxial small-incision cataract surgery (p=0.564). Coma did not significantly change after either of the procedures. Trefoil did not significantly change after 2.2-mm coaxial micro-incision cataract surgery (p=0.361), whereas it significantly increased after 2.75-mm coaxial small-incision cataract surgery (p<0.001). There was no significant difference shown in effective phaco time between the procedures. A significantly positive correlation was shown between surgically induced anterior corneal astigmatism and coma in 2.75-mm coaxial small-incision cataract surgery (r=0.387, p=0.006). There was no significant correlation found between any surgically induced higher-order aberration changes and effective phaco time.

**Conclusions:**

The results showed that 2.2-mm coaxial micro-incision cataract surgery and 2.75-mm coaxial small-incision cataract surgery did not significantly degrade the total higher-order aberrations of the anterior cornea. However, the surgically induced changes in total higher-order aberration showed a significant difference between the two procedures, with a slight reduction after 2.2-mm coaxial micro-incision cataract surgery and a slight increase after 2.75-mm coaxial small-incision cataract surgery. Phaco time and power used during surgery had no impact on corneal aberrations.

## Abstract (pt)

**Objetivo:**

Investigar o impacto de diferentes tamanhos de incisões em córnea clara com meridiano íngreme para facoemulsificação com aberrações de mais alta ordem da córnea anterior.

**Métodos:**

Foram retrospectivamente revisados os prontuários médicos de pacientes que se submeteram a cirurgias de catarata com microincisões coaxiais de 2,2 mm ou com incisões coaxiais pequenas de 2,75 mm. Foram apenas incluídos pacientes com astigmatismo preexistente da córnea anterior <2,00 dioptrias (D) e ≥0,50 D, e submetidos a incisões em córnea clara com meridiano íngreme. Os desfechos primários foram aberrações da córnea anterior da 3ª à 6ª ordem com uma pupila de 8 mm. O astigmatismo da córnea anterior e o tempo efetivo de facoemulsificação foram avaliados como desfechos secundários. Os desfechos pré-operatório e pós-operatório aos 3 meses também foram avaliados.

**Resultados:**

O astigmatismo da córnea anterior diminuiu significativamente após ambos os procedimentos, mas não se encontrou nenhuma diferença significativa entre os dois procedimentos quanto ao astigmatismo da córnea anterior, induzido pela cirurgia (p=0,146). Embora as aberrações totais de mais alta ordem não se tenham alterado significativamente após ambos procedimentos, a comparação entre os grupos revelou uma diferença significativa nas aberrações totais de mais alta ordem, induzidas pela cirurgia (uma diminuição de 0,337 ± 1,156 µm na cirurgia de catarata por microincisão coaxial de 2,2 mm e um aumento de 0,106 ± 0,521 µm na cirurgia de catarata por incisão coaxial pequena de 2,75 mm; p=0,046). A aberração esférica diminuiu significativamente após cirurgia de catarata por microincisão coaxial de 2,2 mm (p=0,001), mas não se alterou significativamente após cirurgia de catarata por incisão coaxial pequena de 2,75 mm (p=0,564). A aberração de coma não mudou significativamente após qualquer dos procedimentos. O trifólio não se alterou significativamente após cirurgia de catarata por microincisão coaxial de 2,2 mm (p=0,361), mas aumentou significativamente após cirurgia de catarata por incisão coaxial pequena de 2,75 mm (p<0,001). Nenhuma diferença significativa se evidenciou quanto ao tempo efetivo de faco-emulsificação entre os dois procedimentos. Houve uma correlação positiva significativa entre o astigmatismo da córnea anterior, induzido pela cirurgia e a aberração de coma na cirurgia de catarata por incisão coaxial pequena de 2,75 mm (r=0,387, p=0,006). Não foi encontrada correlação significativa entre as alterações nas aberrações totais de mais alta ordem, induzidas pela cirurgia e o tempo efetivo de faco-emulsificação.

**Conclusões:**

Nem a cirurgia de catarata por microincisão coaxial de 2,2 mm, nem aquela por incisão coaxial pequena de 2,75 mm degradaram significativamente as aberrações totais de mais alta ordem da córnea anterior. Porém, as alterações nas aberrações totais de mais alta ordem, induzidas pela cirurgia mostraram uma diferença significativa entre os dois procedimentos, com uma ligeira redução na cirurgia de catarata por microincisão coaxial de 2,2 mm e um pequeno aumento na cirurgia de catarata por incisão coaxial pequena de 2,75 mm. O tempo de facoemulsificação e a potência utilizada durante a cirurgia não tiveram impacto nas aberrações corneanas.

## INTRODUCTION

Wavefront aberrations have been commonly used as a robust indicator for assessing optical quality in pseudophakic eyes^(1^-^4)^. Ocular aberrations in pseudophakic eyes are mainly derived from the cornea and intraocular lens (IOLs). Currently, there are various types of commercially available aspheric IOLs aiming to individually compensate for corneal aberrations. However, understanding the changes in corneal aberrations during cataract surgery is an important prerequisite for achieving an optimal optical quality by individually implanting an aspheric IOL. There are currently various sizes of clear corneal incisions (CCIs) that are utilized in cataract surgery. Although the incisions in modern cataract surgery are smaller than those performed in earlier techniques, they continue to induce a change in corneal astigmatism and higher-order aberrations (HOAs) that degrade the optical quality of the cornea^(5)^. Previous studies have suggested that the CCI size^(5^-^8)^ and site^(9^,^10)^ may play an important role in corneal HOAs. Lower-order aberrations should be taken into consideration as a precondition for the optimization of the HOAs. Astigmatism, as one of the most important components of lower-order aberrations, has a significant impact on visual quality in pseudophakic eyes even with a low amount of residual^(11)^. The steep meridian CCI provides a simple, easy to perform, inexpensive, and suitable method for the correction of eyes with a low level of corneal astigmatism^(12)^.

Previous studies on different incision sizes reported limited data using total corneal aberrations^(5^-^8)^ with small and/or medium pupil sizes^(5^-^8)^. In these studies, there was no significant difference shown in corneal HOAs between micro-incision cataract surgery (MICS) and small-incision cataract surgery (SICS)^(5)^, as well as between the different sizes of coaxial MICS (C-MICS) and biaxial MICS^(7)^. In contrast, other studies suggested that surgically induced changes in corneal HOAs were dependent on the incision size^(6^,^8)^. However, some information may be hidden because of the limited effects by previous studies^(13)^. Currently, there is sparse knowledge regarding the effects of the incision size on corneal HOAs when the pupil is >6 mm. In addition, to the best of our knowledge, there was no previous study investigating the effects of different sizes of steep meridian CCIs on corneal HOAs in eyes with low levels of corneal astigmatism (PubMed, year 2000-2019, keywords: “steep meridian” AND “incision size*” AND aberration*). This study aimed to investigate the impact of different sizes of steep meridian CCIs for phacoemulsification on anterior corneal HOAs. This information may be valuable for surgeons to individually select the incision size and aspherical IOLs according to different amounts of preexisting corneal aberrations during cataract surgery, thus optimizing the postoperative visual quality of patients with cataract.

## METHODS

### Study design and participants

This study was approved by the Institutional Review Board of the Affiliated Hospital of North Sichuan Medical College (Nanchong, China). Consecutive medical records of adult patients with cataract who underwent 2.2-mm C-MICS or 2.75-mm coaxial SICS (C-SICS) at The Affiliated Hospital of North Sichuan Medical College between October 2017 and January 2018 were retrospectively reviewed. A total of 48 eyes of 48 patients (24 women and 24 men) in the 2.2-mm group and 50 eyes of 50 patients (29 women and 21 men) in the 2.75-mm group were included in this study. The mean ages in the two groups were 65.63 ± 4.983 years and 67.26 ± 4.81 years, respectively. Only patients whose preexisting anterior corneal astigmatism (ACA) was <2.00 diopters (D) and ≥0.50 D were included. Additional inclusion criteria required that a steep meridian CCI and an aspheric monofocal IOL implantation were performed during surgery. For patients who underwent bilateral cataract surgery, one of the eyes was randomly included. Patients with any other ocular pathologies, including any corneal lesions, uveitis, glaucoma, and retinal and optical nerve diseases, were excluded. Other exclusion criteria that could potentially affect the postoperative vision or the reliability of the study outcome measurements were any intraoperative or postoperative complications, a history of any ocular surgery, or use of a contact lens.

### Surgical procedures

Operations for all included patients were performed by a single experienced surgeon (C.J.L.) under topical anesthesia using the same phacoemulsification system (Infiniti; Alcon Laboratories, USA). The steepest meridian determined through Scheimpflug topography was marked at the corneal limbus with the patient seated upright at the slit lamp. A 2.2-mm or a 2.75-mm CCI was performed at the marked meridian, followed by continuous circular capsulorhexis, hydrodissection, and phacoemulsification cataract extraction. Subsequently, a foldable aspheric monofocal IOL (Acrysof IQ; Alcon Laboratories) was implanted in the capsular bag of all patients.

### Outcome measurements

Data including demographics, axial length, and nuclear density (Emery-Little classification) were extracted and compared at baseline. Primary outcome measurements were 3^rd^- to 6^th^-order anterior corneal HOAs measured using a Scheimpflug imaging system (Pentacam; Oculus Inc., Wetzlar, Germany) with an 8-mm pupil. Secondary outcome measurements included best-corrected visual acuity (BCVA), ACA, and effective phaco time (EPT). ACA was obtained from the Pentacam and determined using the change in simulated keratometry values, which was the difference in power between the steep and flat meridians. Preoperative and 3-month postoperative outcome measurement data were extracted for comparative analyses. Corneal HOAs were compared via categorization into the total HOA (t-HOA), spherical aberration (SA), coma, and trefoil. The t-HOA was denoted as the total root mean square (RMS) of all 3^rd^-6^th^ Zernike coefficients; SA was denoted as the total RMS of 4^th^ (Z_4_^0^) and 6^th^ (Z_6_^0^) SAs; coma was denoted as the total RMS of 3^rd^ (Z_3_^^1^^, Z_3_^-1^) and 5^th^ (Z_5_^^1^^, Z_5_^-1^) coma-like aberrations; trefoil was denoted as the total RMS of 3rd (Z_3_^^3^^, Z_3_^-3^) and 5th (Z_5_^^3^^, Z_5_^-3^) trefoil-like aberrations. ACA was obtained from the corneal topography using the Scheimpflug imaging system. Surgically induced astigmatism was calculated using a vector analysis (Jaffe and Clayman method). The ultrasound time was defined as the time during which the foot pedal remained in position 3. The mean phaco power was defined as the power used during ultrasound time. These two parameters were recorded using the surgical system. The EPT was calculated using the following formula: EPT = ultrasound time (s) × mean phaco power (%)^(14)^.

### Statistical analysis

Sample size calculation was performed on the basis of the results reported by Tong et al.^(8)^, which suggested that a sample of 41 subjects in each group would achieve a power of 80% and a level of significance of 5% (two sided) for the detection of a significant difference in total corneal HOA changes between the two groups. All data were analyzed using the SPSS version 20.0 software analysis system (IBM Corp., Armonk, NY, USA). The Shapiro-Wilk test was used to test the normal distribution of the data. Continuous variables were expressed as the mean ± standard deviation, whereas dichotomous or ordinal variables were expressed in percentages. For normally distributed data, an independent samples t-test was performed for comparisons of means between the two groups; a paired t-test was performed for comparisons of means before and after surgery. Pearson’s correlation test was performed to explore correlations between two variables. For non-normally distributed data, the Mann-Whitney test was performed to compare groups; the Wilcoxon signed-rank test was performed for comparisons before and after surgery; and Spearman’s rank correlation test was performed to explore correlations between two variables. A contingency chi-squared test was performed for dichotomous or ordinal data comparisons between groups. A p-value <0.05 denoted statistically significant differences.

## RESULTS

There was no significant difference in demographics and other baseline characteristics between the two groups (Table 1).

**Table 1:** Demographics and baseline characteristics of the patients

	2.2-mm C-MICS N=48	2.75-mm C-SICS N=50	p-value
Sex (female, %)	50	58	0.427^a^
Laterality (surgery in the right eye, %)	58.3	42	0.106^a^
Nuclear density (% in grades 2, 3, and 4)	25%, 58.3%, 16.7	26, 56, 18	0.971^a^
Age (years)	65.63 ± 4.983	67.26 ± 4.81	0.102^b^
Axial length (mm)	23.413 ± 1.227	24.186 ± 2.515	0.091^c^

a = Chi-squared test;

b = Independent t-test;

c = Mann-Whitney test.

### Comparisons before and after surgery within groups

**Figure 1:**
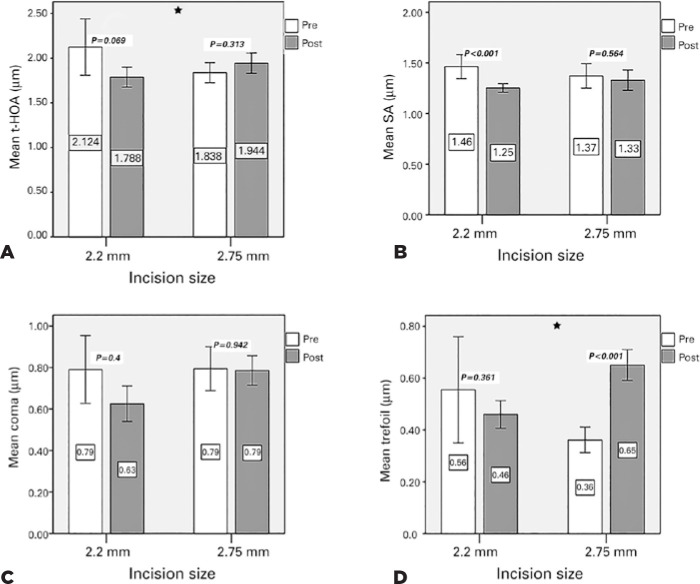
Means and 95% confidence intervals of total anterior corneal higher-order aberration (A), spherical aberration (B), coma (C), and trefoil (D) following 2.2-mm C-MICS and 2.75-mm C-SICS. p-values on the top of the bars show the comparisons before and after surgery. ⛥ The surgically induced changes between groups were significantly different.

As shown in table 2 and figure 1, logarithm of the minimum angle of resolution (logMAR) BCVA was significantly improved after surgery in both groups (p<0.001). ACA significantly decreased after both 2.2-mm C-MICS (p<0.001) and 2.75-mm C-SICS (p<0.001). The corneal t-HOA slightly decreased after 2.2-mm C-MICS and increased after 2.75-mm C-SICS; however, the observed changes in both groups were not statistically significant. The corneal SA decreased significantly after 2.2-mm C-MICS (p=0.001); however, it did not change significantly after 2.75-mm C-SICS. The corneal coma did not change significantly after 2.2-mm C-MICS or 2.75-mm C-SICS. The corneal trefoil after 2.2-mm C-MICS did not change significantly, whereas it significantly increased after 2.75-mm C-SICS (p<0.001).

**Table 2:** Comparisons before and after surgery (mean ± SD)

	2.2-mm C-M1CS			2.75-mm C-S1CS		
	Preoperative	Postoperative	p-value	Preoperative	Postoperative	p-value
BCVA (mean ± SD, logMAR)	1.107 ± 0.961	0.045 ± 0.068	<0.001^*^	1.376 ± 0.862	0.032 ± 0.06	<0.001^*^
ACA (mean ± SD, D)	0.938 ± 0.353	0.473 ± 0.329	<0.001^*^	0.858 ± 0.302	0.539 ± 0.326	<0.001^*^
t-HOA (mean ± SD, µm)	2.124 ± 1.087	1.788 ± 0.381	0.069	1.838 ± 0.394	1.944 ± 0.401	0.313
SA (mean ± SD, µm)^^1^^	1.463 ± 0.406	1.253 ± 0.146	0.001^*^	1.371 ± 0.425	1.33 ± 0.353	0.564
Coma (mean ± SD, µm)	0.79 ± 0.563	0.625 ± 0.294	0.4	0.794 ± 0.374	0.786 ± 0.248	0.942
Trefoil (mean ± SD, µm)	0.555 ± 0.706	0.46 ± 0.185	0.361	0.362 ± 0.173	0.651 ± 0.209	<0.001^*^

* = Statistical significance; ¶= Paired t-test was used; the remaining variables were analyzed using the Wilcoxon signed-rank test.

### Comparisons of surgically induced changes between groups

Table 3 shows that there was no significant difference in BCVA improvement between 2.2-mm C-MICS and 2.75-mm C-SICS. The reductions in ACA after the two procedures were not significantly different. There was a significant difference in surgically induced t-HOA between 2.2-mm C-MICS (decreased by 0.337 ± 1.156 µm) and 2.75-mm C-SICS (increased by 0.106 ± 0.521 µm) (p=0.046). There was no significant difference shown in surgically induced SA and coma between the two procedures. A significant difference was shown in surgically induced trefoil between 2.2-mm C-MICS (decreased by 0.095 ± 0.67 µm) and 2.75-mm C-SICS (increased by 0.289 ± 0.255 µm) (p<0.001). There was no significant difference in the EPT used intraoperatively between the two procedures.

**Table 3:** Comparisons of surgically induced changes and intraoperative EPT (mean ± SD)

	Surgically induced changes^*^		
	2.2-mm C-M1CS	2.75-mm C-S1CS	p-value
BCVA (mean ± SD, logMAR)	1.063 ± 0.979	1.343 ± 0.849	0.079
EPT (mean ± SD, s)	12.578 ± 11.632	19.237 ± 17.129	0.07
ACA (mean ± SD, D)	0.464 ± 0.545	0.32 ± 0.428	0.146^1^
t-HOA (mean ± SD, µm)	0.337 ± 1.156	-0.106 ± 0.521	0.046^*^
SA (mean ± SD, µm)	0.21 ± 0.421	0.041 ± 0.501	0.075^1^
Coma (mean ± SD, µm)	0.165 ± 0.551	0.008 ± 0.459	0.323
Trefoil (mean ± SD, µm)	0.095 ± 0.67	-0.289 ± 0.255	<0.001^*^

* = Statistical significance.

¶ = Independent t-test was used; the remaining data were analyzed using the Mann-Whitney test.

# = For this comparison, negative signs indicate an increase in the variables after surgery; no sign indicates a decrease.

**Correlations between surgically induced anterior corneal HOA changes and ACA changes and EPT**

As shown in table 4, there was no significant correlation found between any corneal HOA component changes and EPT in either of the procedures, except for that observed between t-HOA changes and EPT in 2.75-mm C-SICS (r=-0.28, p=0.048). However, the p-value was at the border of statistical significance, hardly indicating a significant correlation. There was a significantly positive correlation between ACA changes and coma changes in 2.75-mm C-SICS (r=0.387, p=0.006). There was no significant correlation found between ACA changes and any other corneal HOA component changes in either of the procedures (p>0.05).

**Table 4:** Correlations between anterior corneal HOA changes and ACA changes and EPT (correlation coefficient, *r*)

Corneal HOA changes (µm)	2.2-mm C-M1CS				2.75-mm C-S1CS			
	ACA changes (D)	p-value	EPT (s)	p-value	ACA changes (D)	p-value	EPT (s)	p-value
t-HOA	**0.245**	**0.093**	**0.119**	**0.419**	**0.240^§^**	**0.093**	**-0.280**	0.048^*^
SA	**0.065^§^**	**0.66**	**-0.032**	**0.829**	**0.142^§^**	**0.325**	**-0.092**	**0.523**
Coma	**-0.099**	**0.502**	**0.162**	**0.273**	**0.387^§^**	0.006^*^	**-0.058**	**0.687**
Trefoil	**-0.159**	**0.28**	**0.047**	**0.751**	**0.105^§^**	**0.467**	**0.01**	**0.946**

* = Statistical significance;

§ = Pearson’s correlation test was used, and the remaining data were analyzed using Spearman’s correlation test.

## DISCUSSION

In the present study, limited information was improved by using the larger pupil size (8 mm) and anterior corneal surface solely, due to the importance of the greater extent of the optical changes in the mid-periphery of the cornea. Previous studies have shown that the incision size and site have an impact on corneal astigmatism^(15^,^16)^ and HOAs^(6^,^9)^. In the present study, steep meridian CCIs were performed to correct the preexisting corneal astigmatism in all patients. Thus, the incision size was the only interventional variable between groups, indicating that the incision site could not bias our results. It has been suggested that a reduction in corneal astigmatism may be achieved by performing a steep meridian incision, which could be a positive effect of cataract surgery^(17^-^20)^. It has also been suggested that MICS and SICS may exert similar or different effects on cornea wavefront aberrations with small and/or medium pupil sizes^(5^-^8)^. However, there is limited knowledge regarding the influence of the incision size on corneal wavefront aberrations, when a steep meridian incision is performed to correct the low amount of preexisting corneal astigmatism under the condition of a large pupil. This study aimed to determine this influence.

The results of this study suggested that visual acuity improvement after the two procedures was similar. This was consistent with the findings of previous studies related to comparisons between MICS and SICS^(7^,^16)^. Corneal astigmatism significantly decreased after both micro and small steep meridian incisions in this study, further confirming the previous viewpoint that a steep meridian incision may reduce the corneal astigmatism. Reductions in astigmatism after both procedures were similar, indicating that the difference in incision size between 2.2 mm and 2.75 mm steep meridian CCI was not sufficient to result in a significant difference for corneal astigmatism correction. Although the surgically induced astigmatism was similar, the corneal HOA changes differed between the two procedures. As shown in figure 1, corneal HOAs (including t-HOA, SA, coma, and trefoil) decreased after 2.2-mm C-MICS; the decrease in SA was statistically significant. In contrast, all corneal HOA components after 2.75-mm C-SICS were either maintained or increased, and the increase in trefoil was statistically significant. Comparisons of surgically induced corneal HOA changes showed that the 2.2-mm steep meridian incision exhibited superiority over the 2.75-mm steep meridian incision in maintaining or reducing corneal t-HOA and trefoil. The results were consistent with those of previous studies reporting similar superiority of micro-incision over small-incision surgery on surgically induced t-HOA and trefoil^(7^,^8)^. However, compared with previous studies that showed maintained or slightly increased values in HOAs in either MICS or SICS, our study showed a decrease in all HOA components following a 2.2-mm steep meridian C-MICS. This result indicated that performing a 2.2-mm micro-incision at the steep meridian for the correction of even low levels of preexisting corneal astigmatism may be significant for improving the corneal wavefront aberrations. In spite of the significance of astigmatism correction for corneal aberrations shown in our study, most of the surgically induced corneal HOA changes were not significantly correlated with surgically induced corneal astigmatism changes except for coma, which was consistent with the finding reported by Chu et al.^(9)^. The reason for this may be that the effect of steep meridian incision on astigmatism correction is limited. Our results also further confirmed the viewpoint presented by previous studies that the incision played an important role in trefoil aberration^(7^,^8^,^10)^. Even slight enlargements of the incision from 2.2 to 2.75 mm would significantly increase the level of surgically induced trefoil. Because t-HOA mainly consists of SA, coma, and trefoil, the significant difference in surgically induced trefoil may contribute to the difference in surgically induced t-HOA observed between the two procedures.

In this study, the EPTs during the two procedures were similar, which was consistent with the pooled results of a meta-analysis performed by Shentu et al. comparing the EPT between micro-incisions and small incisions^(16)^. This indicated that micro-incisions may render the surgery more challenging; however, they did not increase the energy release or ultrasound time during the surgery. In addition, the EPT was not significantly correlated with any corneal HOA changes, indicating that using ultrasound during phacoemulsification did not significantly affect the corneal optics.

There were some limitations in this study. First, this was a retrospective study. Second, HOAs were measured only in an 8-mm pupil. Finally, the subjective visual performance was not evaluated. Further prospective randomized studies focusing on various pupil sizes and a combination of objective optical quality and subjective visual performance assessment will lead to a stronger evidence-based clinical guidance.

In conclusion, both 2.2-mm steep meridian C-MICS and 2.75-mm steep meridian C-SICS did not degrade the t-HOA of the anterior corneal surface in an 8-mm pupil. However, a micro-incision at steep meridian showed better anterior corneal trefoil and t-HOA. In addition, the smaller incisions do not increase the ultrasound energy and time during the surgery, and the intraoperative ultrasound energy and time had no impact on corneal aberrations. Overall, performing a steep meridian MICS for eyes with a low level of preexisting cor neal astigmatism may be beneficial in maintaining or im proving anterior corneal HOAs. Our results may assist in improving the incision size and IOL selection strategies in cataract surgery, particularly for those with a natural or secondary large pupil.
